# Bacteria in hypertrophic scars promote scar formation through HSBP1‐mediated autophagy

**DOI:** 10.1111/wrr.13253

**Published:** 2025-01-17

**Authors:** Bo Yuan, Jiarong Yu, Jiaoyun Dong, Zhigang Mao, Xiqiao Wang

**Affiliations:** ^1^ Department of Burn, Ruijin Hospital Shanghai Jiao Tong University School of Medicine Shanghai China; ^2^ Department of Plastic Surgery, Ninth People's Hospital Shanghai Jiao Tong University School of Medicine Shanghai China

**Keywords:** autophagy, fibroblasts, HSBP1, hypertrophic scar, *S. aureus*

## Abstract

Bacterial colonisation in hypertrophic scars (HSs) has been reported, yet the precise mechanism of their contribution to scar formation remains elusive. To address this, we examined HS and normal skin (NS) tissues through Gram staining and immunofluorescence. We co‐cultured fibroblasts with heat‐inactivated *Staphylococcus aureus* (*S. aureus*) and evaluated their levels of apoptosis and proliferation by flow cytometry and Cell Counting Kit‐8 assay, respectively. Additionally, we performed proteomic analysis and western blotting to identify upregulated proteins. To assess autophagy levels, we examined light chain 3 (LC3) expression through western blotting and immunofluorescence, and transmission electron microscopy (TEM) was performed to detect autophagy‐associated vesicles. Our results demonstrated a notable increase in bacterial load, primarily *S. aureus*, in HS tissues. Furthermore, *S. aureus* promoted fibroblast proliferation and enhanced the expression of profibrotic markers such as transforming growth factor β1 (TGF‐β1), vascular endothelial growth factor (VEGF), collagen I, collagen III and α smooth muscle actin (α‐SMA). Proteomic analysis highlighted heat shock factor‐binding protein 1 (HSBP1) as a key upregulated protein mediating the profibrotic effects induced by *S. aureus*. Knockdown of HSBP1 reversed these effects. Intriguingly, HSBP1 also upregulated LC3 and Beclin‐1 expression and increased the number of autophagosomes in fibroblasts. Finally, when fibroblasts stimulated by *S. aureus* were treated with HSBP1 siRNA, autophagy levels decreased significantly. Collectively, our findings suggest that *S. aureus*, via HSBP1, stimulates fibroblast proliferation and promotes their transition into myofibroblasts, triggering autophagy and fibrosis. These results underscore the potential of HSBP1 as a therapeutic target for the management of HSs.

## 1 INTRODUCTION

Human hypertrophic scars (HSs) frequently arise following severe skin trauma. Characterised by an abnormal accumulation of extracellular matrix (ECM), these scars often lead to both aesthetic deformities and functional impairments and can pose a significant clinical challenge.^1^, ^2^, ^3^, ^4^ Although there are many modalities to treat HSs, the effectiveness of these methods is limited, especially for scars resulting from larger burn areas. Consequently, it is imperative to devise innovative approaches that aim to promote non‐scarring or scarless healing; therefore, a deeper understanding of the fundamental mechanisms driving this process is essential.

Previously, scars were believed to be devoid of bacteria after wound healing. However, our recent study has uncovered the presence of excessive bacterial colonisation in HSs, predominantly by *Staphylococcus aureus*.^5^ This discovery suggests a potential link between bacterial colonisation and the formation of HSs. Notably, *S. aureus* is the most frequently encountered pathogen and plays pivotal roles in numerous inflammatory diseases, such as atopic dermatitis, psoriasis, pulmonary cystic fibrosis, allergic asthma and osteomyelitis,^6^ hinting at its potential involvement in scar development. In pulmonary cystic fibrosis, *S. aureus* infects the patient's airways, resulting in chronic inflammation and poor prognosis.^7^ However, the mechanism by which *S. aureus* causes fibrosis is not clear.

Heat shock proteins (HSPs) are major stress‐inducible proteins that play a crucial role in the response to bacterial infection. Studies have demonstrated that when human monocytes are infected with the vaccine virus or *S. aureus*, they activate multiple cellular anti‐apoptotic and cytoprotective mechanisms to enhance the viability and proinflammatory activity of monocyte–macrophage lineage cells. For instance, macrophages exhibit increased expression of HSP 1, leading to prolonged inflammation.^8^ HSBP1 serves as a binding protein for HSP, exerting a negative regulatory effect on the functions of HSPs.^9^ It has been discovered that HSBP1 modulates the proliferation, differentiation and invasion of tumour cells, thereby substantially enhancing the tumour's invasive capacity.^10^ However, as a member of the heat shock system, whether HSBP1 can respond to bacterial stimulation and its relationship with fibrosis have not been studied.

Thus, in this study, we aim to investigate the mechanism by which bacteria induce scar formation and explore the possible role of HSBP1 during this process.

## 2 MATERIALS AND METHODS

### 2.1 Collection of HS and NS samples

A total of eight patients, aged 21 ± 4.2 years, presenting with HSs on their limbs or trunks, were recruited for this study, which was conducted in the Burn Department of Rui Jin Hospital, Shanghai Jiao Tong University, from January 2018 to April 2021. These scars, ranging in duration from 10 to 15 months, exhibited characteristics such as elevation, redness and hardness, necessitating scar excision and skin transplantation. Following skin transplantation, normal skin (NS) samples were collected from the donor sites, primarily the abdomen and served as controls for the study. All patients provided their informed consent prior to participation.

### 2.2 Tissue processing for histochemistry

Tissue samples were initially fixed in 10% buffered formalin for a minimum duration of 48 h. Subsequently, the tissues underwent overnight processing in gradually increasing concentrations of ethanol. This was followed by three thorough washes in xylene and subsequent equilibration in paraffin. Once prepared, the tissues were embedded in paraffin blocks and then precisely cut into 6‐μm‐thick sections. To prepare the slides for further analysis, they were first deparaffinized and cleared using xylene, followed by rehydration procedures that were tailored for Gram staining and immunohistochemical procedures.

### 2.3 Gram staining

Gram staining was performed according to the manufacturer's protocol (Beijing Solarbio Science & Technology, Beijing, China). First, crystal violet was added to the tissue on the slide, which stained all the bacteria purple. Then, Gram's iodine was used to increase the retention of the purple colour. Alcohol was then used to wash away part of the purple colour, depending on the thickness of the bacterial cell walls. After rinsing off the alcohol, safranin was added to stain the bacteria from which the purple colour had been rinsed away a pink colour. Two types of bacteria could then be observed: purple‐stained cells (Gram‐positive) and pink‐stained cells (Gram‐negative).

### 2.4 Immunofluorescence for *S. aureus*

The tissue sections were first deparaffinized, rehydrated and rinsed thoroughly in distilled water. The slides were then blocked with 3% bovine serum albumin (BSA) to prevent non‐specific binding. Following this, the slides were incubated with a rabbit polyclonal anti‐*S. aureus* antibody (1:100, Abcam, catalogue number ab20920) overnight at 4°C. Subsequently, the slides were washed three times with phosphate‐buffered saline (PBS) to remove unbound antibodies. Next, the slides were incubated with donkey anti‐rabbit IgG secondary antibody conjugated to Alexa Fluor 488 (1:500, Thermo Fisher Scientific, catalogue number A21206) for 1 h at room temperature, which allowed for the visualisation of the bound primary antibodies. To stain the cell nuclei, the slides were incubated with 4′,6‐diamidino‐2‐phenylindole (DAPI, 1:1000, Cell Signalling Technology, catalogue number 4083) for an appropriate duration. Following three additional washes, the slides were mounted and observed under a fluorescence microscope for imaging.

### 2.5 Isolation and culture of fibroblasts from NS tissue

NS dermal fibroblasts were isolated and cultured in Dulbecco's modified Eagle medium (DMEM) as previously described.^11^ Specifically, full‐thickness skin from the abdominal wall was obtained from redundant skin tissue during plastic surgery. Using a scalpel, the upper epidermal and superficial dermal layers were carefully removed to ensure that primarily fibroblasts derived from the deep dermis were utilised in this study. After the cells reached 80% confluence, they were used for subsequent experiments.

### 2.6 *S. aureus* culture and inactivation

Strains of *S. aureus* were procured from Beina Chuanglian Biology Research Institute (Beijing, China). Bacteria were cultured in nutrient broth medium for 24 h at 37°C and quantified using the colony counting method to obtain concentrations of 10^4^, 10^5^ and 10^6^ colony‐forming units (CFU)/mL in DMEM (Gibco, Life Technologies, USA). The bacteria were then inactivated at 70°C for 30 min. No bacterial colonies were observed when heat‐inactivated bacteria were plated on agarose plates overnight at 37°C.

### 2.7 Cell treatment with bacteria

NS fibroblasts were treated with 10^4^, 10^5^ and 10^6^ CFU/mL inactive *S. aureus*. After 24 h, the cells were collected and subjected to a reverse transcription‐quantitative polymerase chain reaction (RT‐qPCR), western blot and α smooth muscle actin (α‐SMA) immunofluorescence and proteomic analysis, respectively.

### 2.8 Lentivirus transfection

Lv‐HSBP1 lentiviruses were purchased from OBiO Technology (Shanghai, China). Cells were transfected at 30%–50% confluence. After 12 h, >95% of cells were viable. The medium was changed, and cells were passaged after 3 days. After storing a portion of the cells, the remaining cells were used for further experiments. The transfection efficiency was assessed by western blotting.

### 2.9 siRNA transfection

siRNAs against HSBP1 (si‐HSBP1) were acquired from OBiO Technology. Cells were inoculated and cultured in six‐well plates for 24 h to achieve a cell density of 60%–70%. Next, 50 nM of control or siRNA duplexes were added according to instructions provided for the Lipofectamine 3000 siRNA transfection system (Thermo Fisher Scientific, Waltham, MA, USA). The control group used a universal negative control that was non‐homologous to the target gene sequence and exerted no interference on the host genes. The gene knockdown effect was verified by RT‐qPCR.

### 2.10 Flow cytometry for apoptosis

Fibroblasts were treated with *S. aureus* or siRNAs and were collected. Then, 300 μL Annexin V‐fluorescein isothiocyanate (FITC) binding solution, 10 μL Annexin V‐FITC and 10 μL propidium iodide (PI) were added (Yeason, China). The mixture was incubated at room temperature and in the dark for 30 min. The cells were then resuspended in a cell staining buffer for flow cytometry. Staining was assessed on an LSRFortessa flow cytometer (BD Biosciences), and the results were analysed using FlowJo 10 software (FlowJo LLC).

### 2.11 RT‐qPCR

Total RNA was isolated from fibroblasts using TRIzol. cDNA was synthesised using a HiScript® III‐RT SuperMix kit (Vazyme, R323‐01). The cDNA samples were subjected to RT‐qPCR on an ABI 7500 Sequence Detection System (Thermo Fisher). The PCR conditions were as follows: initial denaturation for 10 min at 95°C, followed by 40 cycles of 15 s at 95°C, 15 s at 58°C and 40 s at 72°C. Fold changes in gene expression relative to the normal control were calculated with the DeltaDeltaCT method. Primers were designed using Primer Express software. Absolute quantitative mRNA levels were calculated using standard curves as previously described. All procedures were performed according to manufacturer protocols.^12^ The primer sequences are listed in Table 1.

**TABLE 1:** Primers used in the RT‐qPCR assay.

Gene	Organisms	Forward (5′‐3′)	Reverse (5′‐3′)
GAPDH	Homo sapiens	ACAACTTTGGTATCGTGGAAGG	GCCATCACGCCACAGTTTC
LC3	Homo sapiens	AACATGAGCGAGTTGGTCAAG	GCTCGTAGATGTCCGCGAT
Beclin	Homo sapiens	CCATGCAGGTGAGCTTCGT	GAATCTGCGAGAGACACCATC

### 2.12 Western blotting

Western blotting was performed according to a standard protocol. The details of the primary and secondary antibodies used are listed in Table 2. Target protein band intensities were measured using ImageJ software and standardised against glyceraldehyde‐3‐phosphate dehydrogenase (GAPDH).

**TABLE 2:** Antibodies for western blot.

Primary antibody	Working dilution	Vendor	Catalogue
TGF‐β1	1:1000	Abcam, Cambridge, MA, USA	ab215715
VEGF	1:1000	Cell Signalling Technology, MA, USA	50,661
Collagen I	1:1000	Abcam, Cambridge, MA, USA	ab138492
Collagen III	1:1000	Abcam, Cambridge, MA, USA	ab184993
HSBP1	1:1000	Proteintech	10,169‐2‐AP
LC3A/B	1:1000	Cell Signalling Technology, MA, USA	12,741
Beclin‐1	1:1000	Cell Signalling Technology, MA, USA	3738
GAPDH	1:1000	Cell Signalling Technology, MA, USA	2118

### 2.13 Immunofluorescence for α‐SMA and light chain 3 (LC3)

Cell slides were fixed with paraformaldehyde for 10 min. Slides were washed with PBS three times and permeabilised with 0.1% Triton X‐100. Both tissue slides and cell slides were incubated with 3% BSA (Beyotime, China) in PBS to minimise non‐specific binding of the primary antibody and subsequently incubated with primary antibodies (Abcam, Cambridge, UK; α‐SMA: ab5694; LC3: ab232940) overnight at 4°C. After washing, slides were treated with a secondary antibody for 1 h at 25°C. The nuclei were counterstained with DAPI, and images were captured under a confocal fluorescence microscope. All experiments were performed in triplicate.

### 2.14 Proteomic analysis

The processes to prepare samples for proteomics analysis included protein digestion, isobaric tagging for relative and absolute quantitation (iTRAQ) labelling, strong cation exchange fractionation, liquid chromatography (LC)–mass spectrometry (MS)/MS analysis, protein identification and protein quantitation. Briefly, trypsin digestion and iTRAQ labelling were performed. Then, the mixed peptides were fractionated by strong cation exchange chromatography on an ultimate high‐performance LC system. MS analysis of the iTRAQ‐labelled samples was performed on a QExactive LC–MS/MS Mass Spectrometer (Thermo Fisher Scientific), with three independent MS/MS runs performed for each sample. The Proteome Discoverer software program (Thermo Fisher Scientific) was used for data acquisition and quantification.

Proteins with quantification *p* values <0.05 and fold changes >1.2 were identified as differentially expressed proteins. Functional classification of differentially expressed proteins was performed using Gene Ontology (GO) analysis (https://biit.cs.ut.ee/gprofiler/gost), which groups differentially expressed genes into three categories: biological processes, molecular functions and cellular components. The results were visualised using Hiplot (https://hiplot.com.cn/).

### 2.15 Transmission electron microscopy (TEM)

A sterile polystyrene film was spread at the bottom of a six‐well plate, followed by cell paving. After 24 h, the experimental group was treated with 5 × 10^3^ *S. aureus* and incubated for 6 h. TEM samples were then prepared as described previously.^5^ Briefly, the cell patches were fixed, dehydrated, infiltrated, embedded, polymerised, sectioned and stained with 3% uranyl acetate in 70% methanol and 30% water for 7 min, followed by staining with lead citrate for 10 min. The samples were then viewed at 120 kV in a FEI (TALOS L 120C) electron microscope.

### 2.16 Statistical analysis

Statistical evaluations and graphical representations for the haematoxylin and eosin staining, Gram staining, immunohistochemistry, enzyme‐linked immunosorbent assay (ELISA) and western blotting analyses were conducted using GraphPad Prism 8 software. For statistical analysis, either a one‐way ANOVA followed by Tukey's post hoc multiple comparison test or the Kruskal–Wallis Test accompanied by Dunn's multiple comparison procedure was employed, depending on the data's distribution and other statistical assumptions.

## 3 RESULTS

### 3.1 High counts of *S. aureus* colonised in HSs

**FIGURE 1:**
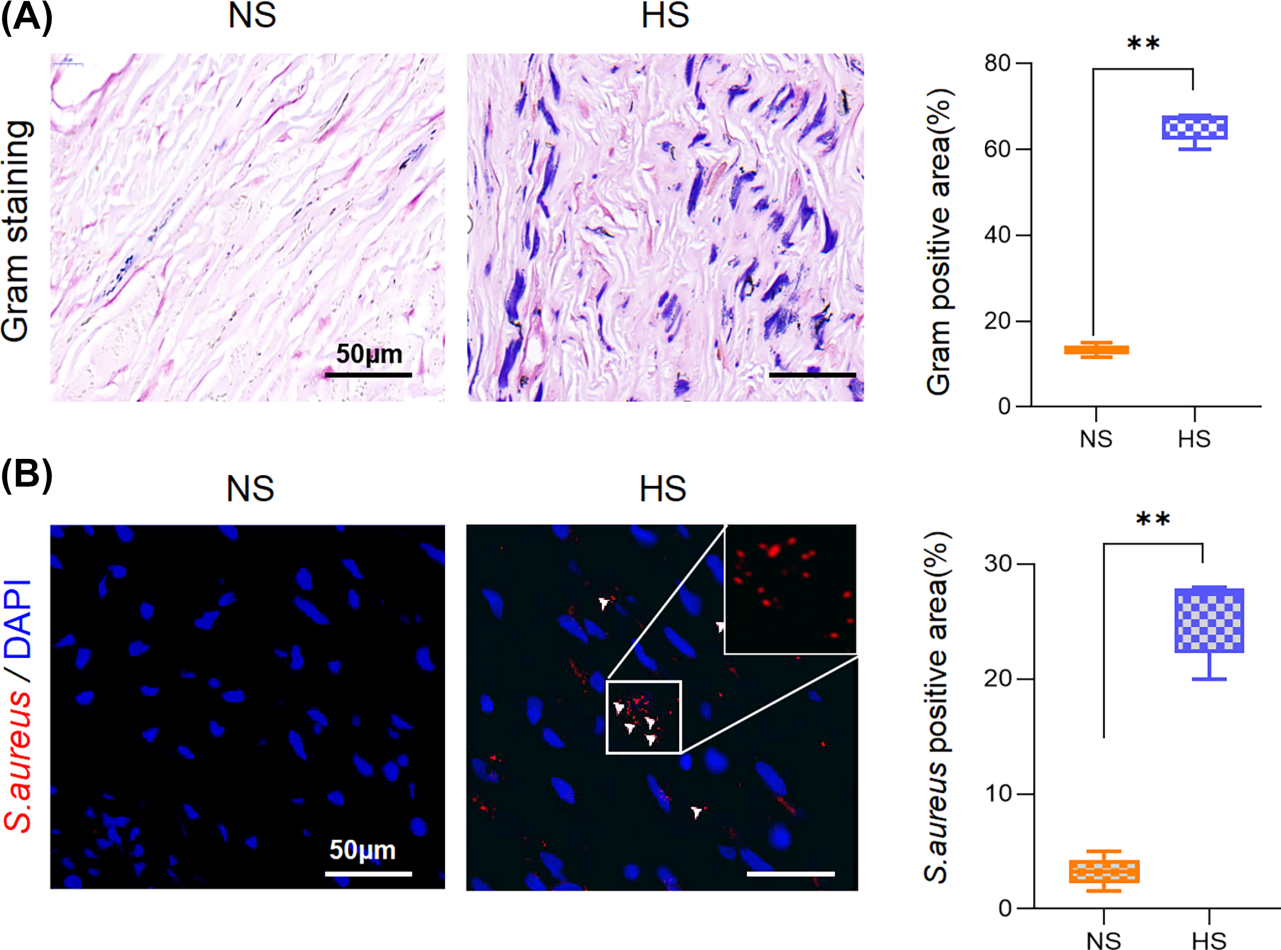
High count of *S. aureus* colonise in HS. (A) Representative Gram staining of NS and HS tissue. The relative gram‐positive area was calculated by imageJ and represented as mean ± SD. Scale bar: 50 μm. **p < 0.01, *n* = 6. (B) Representative immunofluorescence microscopy demonstrating *S.aureus* colonisation in NS and HS tissue. The relative *S. aureus‐*positive account was calculated by imageJ and represented as mean ± SD. Scale bar: 50 μm. **p < 0.01, *n* = 6. Statistical analysis was performed using one‐way ANOVA with Tukey's multiple comparison test.

In this study, we first applied Gram staining to detect bacteria in HS and NS tissue samples. Consistent with our previous findings, the results indicated a significant presence of Gram‐positive bacteria in HS tissue, whereas the bacteria were nearly absent in NS tissue (Figure 1A). To ensure which kind of Gram‐positive bacteria colonised the samples, immunofluorescence staining using an S. aureus‐specific antibody was performed, and the results revealed a markedly elevated content of *S. aureus* in HS tissue compared with NS tissue, reinforcing the observation that a substantial amount of *S. aureus* bacteria colonises HS tissue (Figure 1B).

### 3.2 *S. aureus* regulates apoptosis, cell viability and the myofibroblast (MFB) transition to fibroblasts

To elucidate the mechanisms by which *S. aureus* modulates apoptosis and the differentiation of fibroblasts, we isolated and identified *S. aureus* from HS tissue and subsequently co‐cultured human fibroblasts (HFBs) with varying concentrations of inactivated *S. aureus*.

**FIGURE 2:**
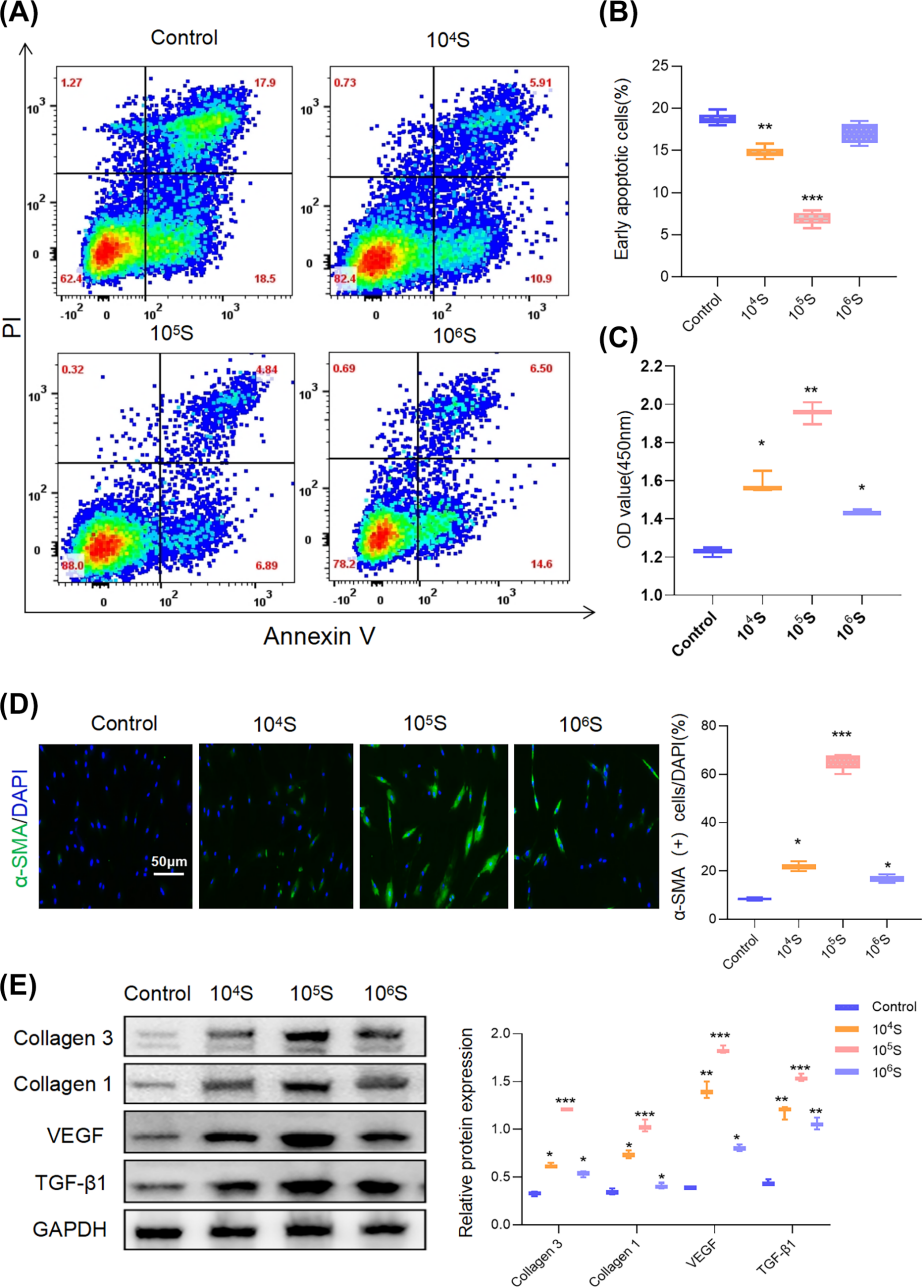
*S. aureus* regulates apoptosis, cell viability and fibroblasts to myofibroblasts transition in HFBs. (A, B) After fibroblasts were stimulated by different concentrations of *S. aureus*, early apoptotic cells were detected by flow cytometry. Graphs were expressed as mean ± SD. Statistical analysis was performed using one‐way ANOVA with Tukey's multiple comparison test. **p < 0.01, ***p < 0.001, *n* = 3. (C) CCK8 was performed to detect the proliferation of normal fibroblasts after being treated with *S. aureus* in the concentration of 10^4^, 10^5^ and 10^6^ CFU/mL. All tests were performed at least three times. Graphs were expressed as mean ± SD. Statistical analysis was performed using one‐way ANOVA with Tukey's multiple comparison test. *p < 0.05, **p < 0.01, *n* = 3. (D) Representative immunofluorescence microscopy demonstrating α‐SMA expression in fibroblasts treated with *S. aureus* in the concentrations of 10^4^, 10^5^ and 10^6^ CFU/mL. Scale bar:50 μm. Statistical analysis was performed using one‐way ANOVA with Tukey's multiple comparison test. *p < 0.05, ***p < 0.001, *n* = 3. (E) Western blot analysis of Collagen3, Collagen1, VEGF and TGF‐β1 in fibroblasts treated with *S. aureus* in the concentration of 10^4^, 10^5^ and 10^6^ CFU/mL. The protein levels were normalised to GAPDH levels. Graphs are presented as mean ± SD. Data were analysed using a two‐way ANOVA with Dunnett's multiple comparison test. *p < 0.05, **p < 0.01, ***p < 0.001, *n* = 3.

The exposure of HFBs to *S. aureus* stimulation led to a significant reduction in apoptosis, as evaluated by flow cytometry, in comparison to untreated controls. Notably, the inhibitory effect of *S. aureus* on apoptosis exhibited a concentration‐dependent pattern, with the most pronounced effect observed at a concentration of 10^5^ CFU/mL (Figure 2A,B). Subsequently, we assessed the impact of *S. aureus* on the viability of HFBs in a CCK‐8 assay. The findings indicated that *S. aureus* promoted HFB viability compared to the control group, with the most pronounced effect occurring at a concentration of 10^5^ CFU/mL (Figure 2C).

Immunofluorescence was performed to detect the expression of α‐SMA, a specific marker for MFBs, in fibroblasts.^13^ Our observations revealed that the expression of α‐SMA increased in response to 10^4^ and 10^5^ CFU/mL of *S. aureus* treatment; however, interestingly, this effect was reversed upon treatment with a higher concentration of 10^6^ CFU/mL, which resulted in a decrease in α‐SMA expression (Figure 2D). Similar patterns were also discernible in the expression levels of transforming growth factor β1 (TGF‐β1), vascular endothelial growth factor (VEGF), collagen 1 and collagen 3, which were detected by western blotting (Figure 2E). Collectively, these results underscore the capacity of *S. aureus* to stimulate the proliferation of HFBs and induce MFB transition at lower concentrations.

### 3.3 HSBP1 is highly expressed in *S. aureus*‐treated HFB

**FIGURE 3:**
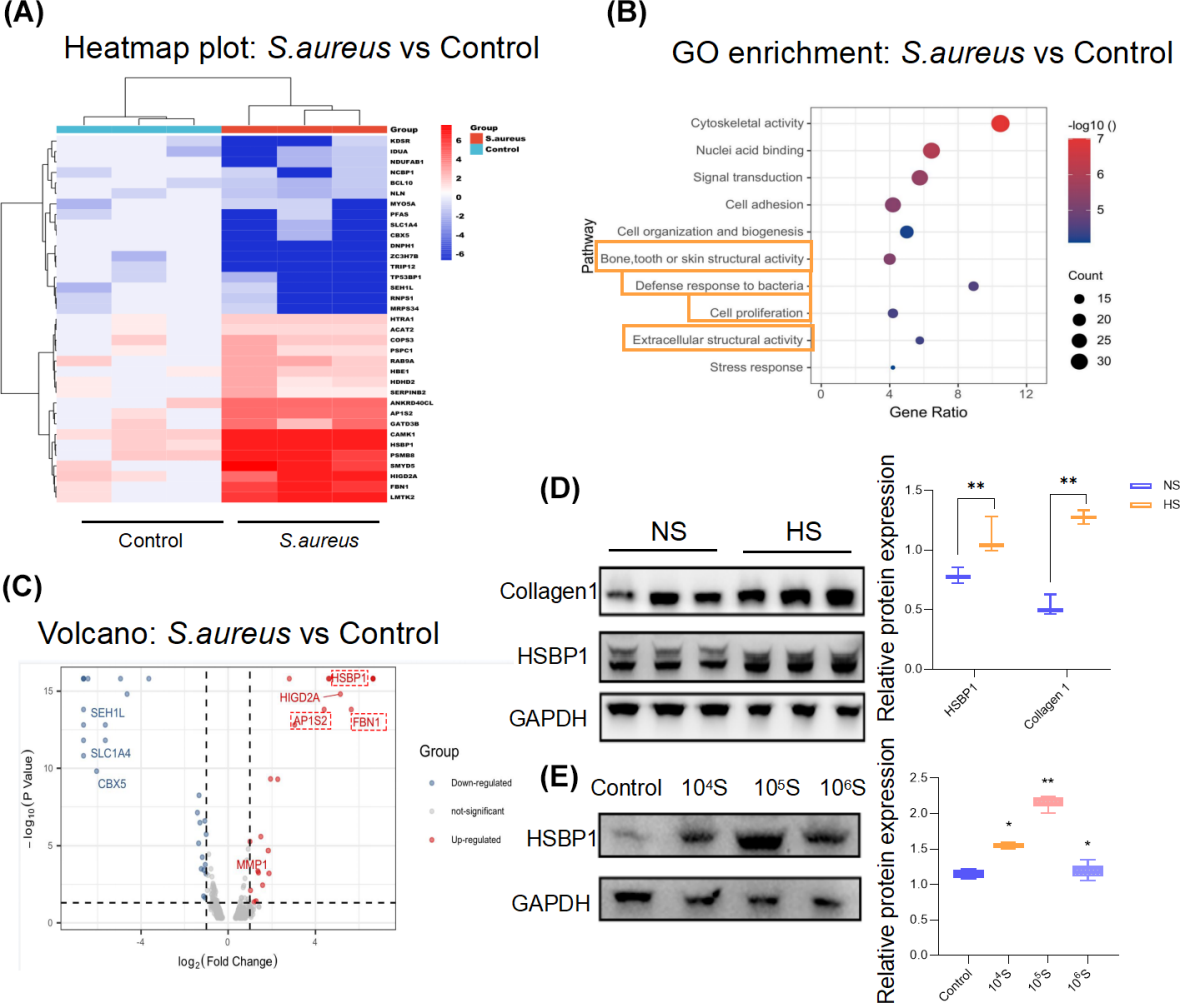
HSBP1 is highly expressed in *S. aureus*‐treated HFB. (A) Protein expression heatmap of significantly up‐ or down‐regulated genes between the treated group and control group. *p* < 0.01, *n* = 3. (B) GO enrichment for changing proteins in *S. aureus* treated fibroblasts revealed their correlation with extracellular structural activity and bacteria defensive activity. (C) Volcano plot showed significantly up‐ or down‐regulated proteins between the *S. aureus*‐treated group and control group. The top highly expressed proteins were enclosed in a red box. (D) Western blot analysis of HSBP1 and Collagen1 in NS and HS tissue. The protein levels were normalised to GAPDH levels. Graphs are presented as mean ± SD. Statistical analysis was performed using one‐way ANOVA with Tukey's multiple comparison test. **p < 0.01, *n* = 3. (E) Western blot analysis of HSBP1 in fibroblasts treated with *S. aureus* in the concentration of 10^4^, 10^5^ and 10^6^ CFU/mL. The protein levels were normalised to GAPDH levels. Graphs are presented as mean ± SD. Statistical analysis was performed using one‐way ANOVA with Tukey's multiple comparison test. *p < 0.05, **p < 0.01, *n* = 3.

To obtain a comprehensive understanding of the proteomic landscape of fibroblasts in response to *S. aureus*, a label‐free quantitative proteomics analysis was conducted using LC/MS. The heatmap generated from this analysis revealed distinct protein expression patterns between the *S. aureus*‐treated group and the untreated group, implying that *S. aureus* exerts varied and significant impacts on the biology of fibroblasts (Figure 3A). Furthermore, GO enrichment analysis highlighted the top 10 biological pathways that were enriched in the *S. aureus*‐treated group. These pathways encompassed cell proliferation, extracellular structural organisation and autophagy, among others, indicating that *S. aureus* modulates fibroblasts through these critical cellular processes (Figure 3B).

Based on the volcano plot analysis, the most prominently upregulated protein in the *S. aureus*‐stimulated group, which exhibited a log2 fold change greater than 2, was identified as HSBP1 (Figure 3C). Western blotting analysis and subsequent quantitative assessments validated the upregulation of HSBP1 in both HS tissue and *S. aureus*‐treated HFBs (Figure 3D,E).

It should be noted that in addition to HSBP‐1, the expression levels of hypoxia‐inducible gene domain family member 2A (HIGD2A), adapter‐related protein complex 1 subunit sigma 2 (AP1S2) and fibrillin‐1 (FBN1) also increased. HIGD2A is an inner mitochondrial membrane protein that increases cell survival under hypoxia. AP1 has been reported to participate in the inflammatory process. FBN1 is an ECM glycoprotein that serves as a structural component. All these upregulated proteins are correlated with inflammation and fibrosis.

### 3.4 *S. aureus*‐mediated promotion of the fibrosis‐related phenotype in HFBs is dependent on HSBP1

**FIGURE 4:**
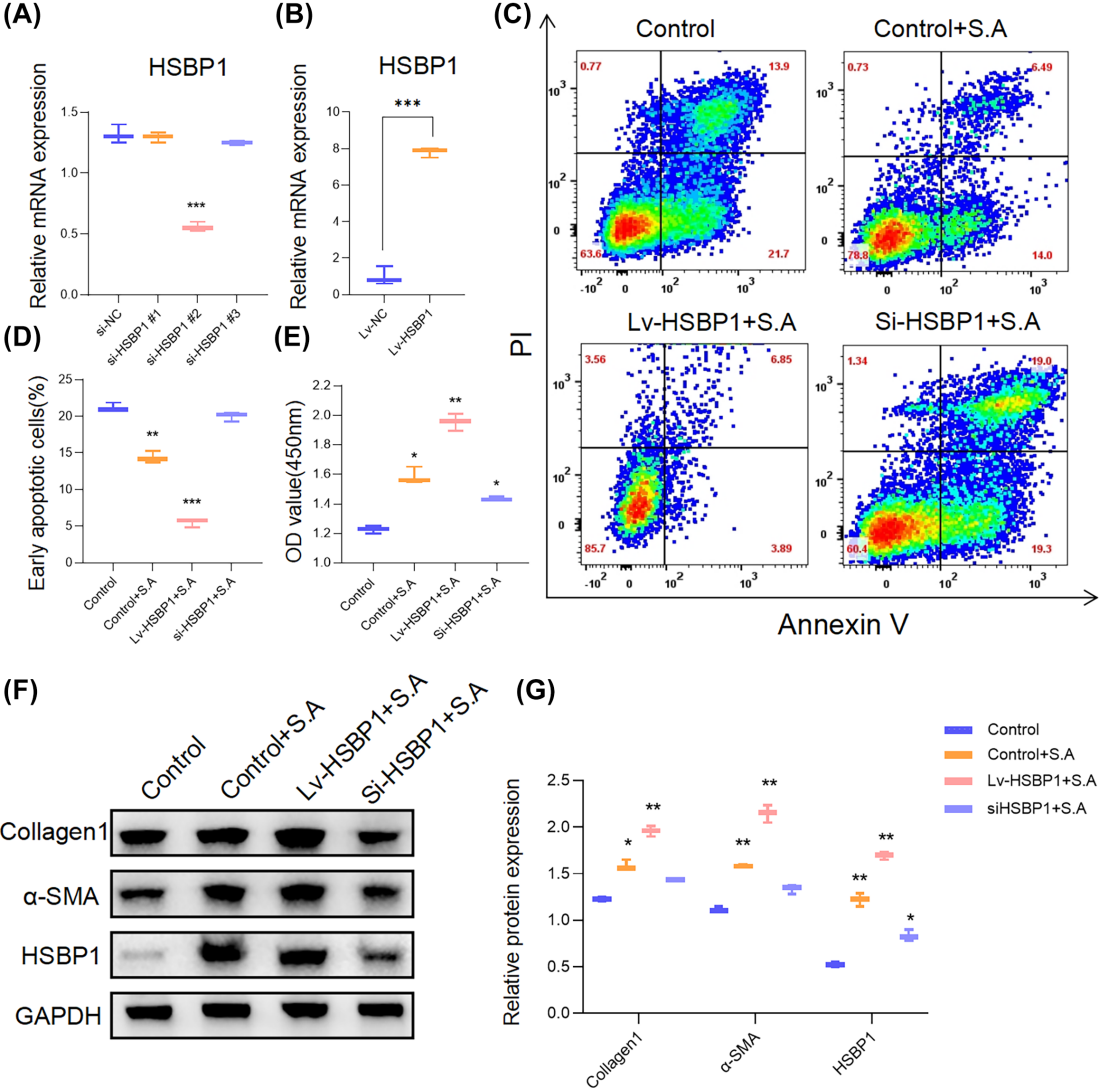
The regulation of *S. aureus* on HFB fibrosis is HSBP1‐dependent. (A) The knockdown efficiency of HSBP1 in fibroblasts detected by qRT‐PCR. ***p < 0.001, *n* = 3. (B) The overexpression efficiency of HSBP1 in fibroblasts detected by qRT‐PCR. ***p < 0.001, *n* = 3. (C, D) The fibroblasts were treated with siHSBP1 and Lv‐HSBP1, and the early apoptotic cells were detected by flow cytometry. Graphs were expressed as mean ± SD. **p < 0.01, ***p < 0.001, *n* = 3. (E) CCK8 was performed to detect the proliferation of fibroblasts after being treated with siHSBP1 and Lv‐HSBP1. All tests were performed at least three times. Graphs were expressed as mean ± SD. *p < 0.05, **p < 0.01, *n* = 6. Statistical analysis was performed using one‐way ANOVA with Tukey's multiple comparison test.(F, G) Western blot analysis of collagen1, α‐SMA and HSBP1 in fibroblasts treated with Lv‐HSBP1 or si‐HSBP1. The protein levels were normalised to GAPDH levels. Graphs are presented as mean ± SD. Data were analysed using a two‐way ANOVA with Dunnett's multiple comparison test. *p < 0.05, **p < 0.01, *n* = 3.

To investigate whether *S. aureus* promotes fibrosis through its interaction with HSBP1, we manipulated HSBP1 expression levels in HFBs using three HSBP1‐specific siRNAs for knockdown and a lentiviral vector for overexpression. RT‐qPCR analysis verified the successful knockdown and overexpression of HSBP1, establishing a functional platform for studying the role of HSBP1 in *S. aureus*‐induced fibrosis (Figure 4A). By co‐infecting HFBs with a lentiviral vector overexpressing HSBP1 (Lv‐HSBP1) and HSBP1‐specific siRNAs (si‐HSBP1), we observed that the downregulation of HSBP1 mitigated the reduction in cell apoptosis induced by *S. aureus*. This resulted in a proliferation rate that was comparable to that of the control group (Figure 4B,C). Furthermore, the elevated MFB transition induced by *S. aureus* was reversed in the si‐HSBP1 group, as evidenced by significant decreases in the protein levels of collagen 1, collagen 3 and α‐SMA compared to HFBs infected with *S. aureus* alone (Figure 4D). However, the effects of *S. aureus* on apoptosis, cell viability and MFB transition were further enhanced by HSBP1 overexpression (Figure 4B–D). Overall, these findings indicate that *S. aureus* exerts its regulatory effects on apoptosis and MFB transition in HFBs through the targeting of HSBP1, thereby modulating cellular processes associated with fibrosis.

### 3.5 HSBP1 enhances autophagy in *S. aureus*‐treated HFBs

**FIGURE 5:**
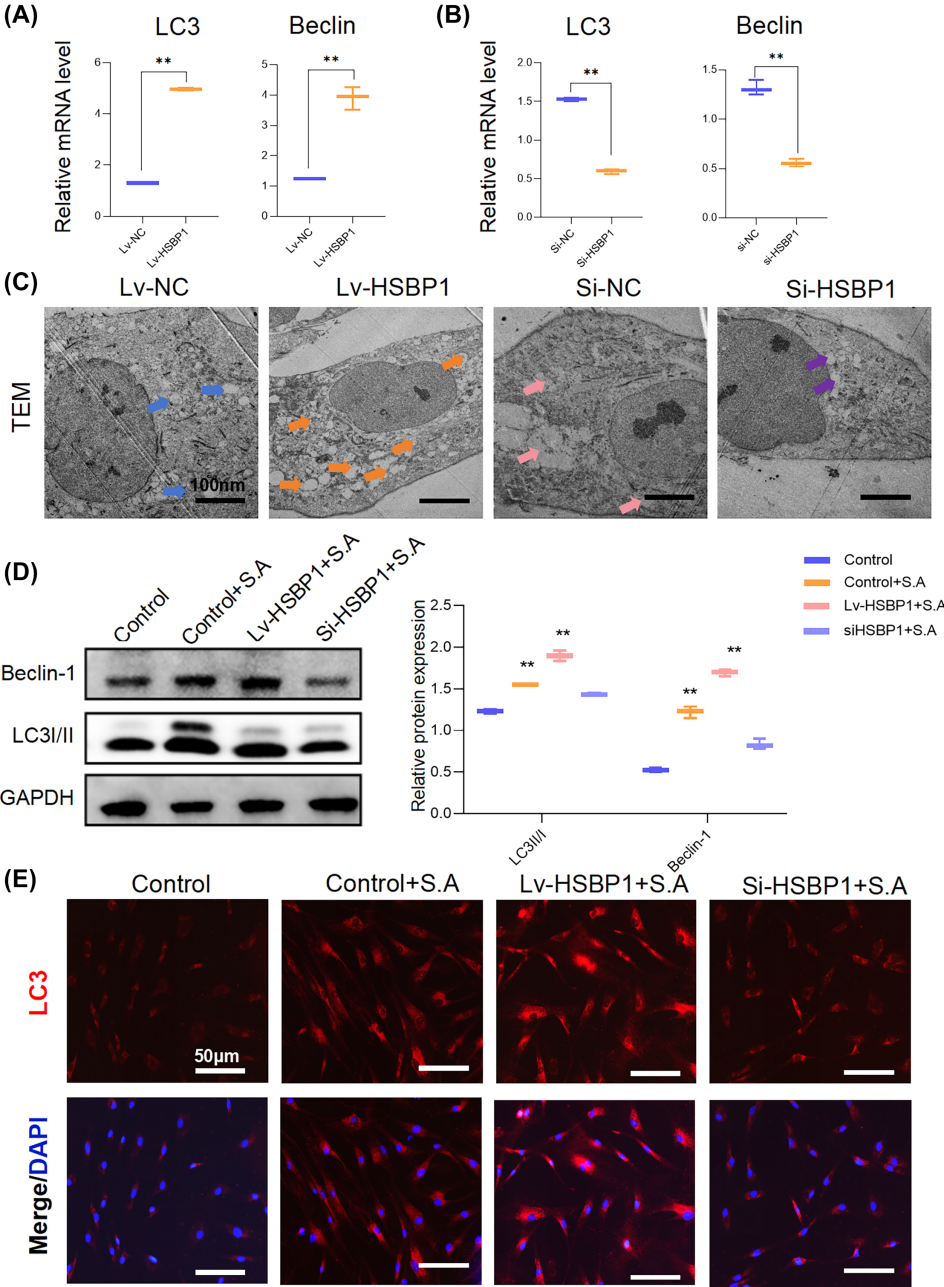
HSBP1 enhances autophagy in *S. aureus*‐treated HFB. (A) The expression levels of Beclin1 and LC3 were detected by qRT‐PCR when HSBP1 was overexpressed in fibroblasts. **p < 0.01, *n* = 3. (B) The expression levels of Beclin1 and LC3 were detected by qRT‐PCR when HSBP1 was downregulated in fibroblasts. **p < 0.01, *n* = 3. Statistical analysis was performed using one‐way ANOVA with Tukey's multiple comparison test. (C) Representative TEM images of fibroblasts treated with Lv‐HSBP1 or si‐HSBP1. Scale bar, 100 nm, *n* = 6. (D) Western blot analysis of Beclin1 and LC3 in fibroblasts treated with Lv‐HSBP1 or si‐HSBP1. The protein levels were normalised to GAPDH levels. Graphs are presented as mean ± SD. Data were analysed using a two‐way ANOVA with Dunnett's multiple comparison test. **p < 0.01, *n* = 3. (E) Representative immunofluorescence microscopy demonstrating LC3 expression in fibroblasts treated with Lv‐HSBP1 or si‐HSBP1. Scale bar, 50 μm, *n* = 6.

Next, we examined whether HSBP1 regulates autophagy. RT‐qPCR analysis revealed that HFBs treated with siHSBP1 expressed lower levels of autophagy markers, such as Beclin1 and LC3, compared to untreated controls (Figure 5A). TEM analysis provided further confirmation, revealing a decrease in the number of autophagy‐associated vesicles in HFBs treated with siHSBP1 compared to controls (Figure 5C). In contrast to the downregulation of HSBP1, overexpression of HSBP1 in HFBs not only led to enhanced expression levels of autophagy markers such as Beclin1 and LC3 but also promoted the formation of autophagy‐related vesicles, as visualised by electron microscopy (Figure 5B,C). Furthermore, western blotting analysis demonstrated that *S. aureus* stimulation significantly upregulated the expression of autophagy markers, including Beclin1 and LC3, in HFBs. Notably, the stimulatory effect of *S. aureus* on autophagy was further augmented in cells overexpressing HSBP1. Conversely, in fibroblasts in which HSBP1 expression was knocked down, *S. aureus* failed to exert its influence on autophagy (Figure 5D). The expression of LC3 was further verified by immunofluorescence (Figure 5E).

### 3.6 Autophagy regulates an *S. aureus*‐induced HFB fibrosis‐related phenotype except for HSBP1‐mediated pathways

**FIGURE 6:**
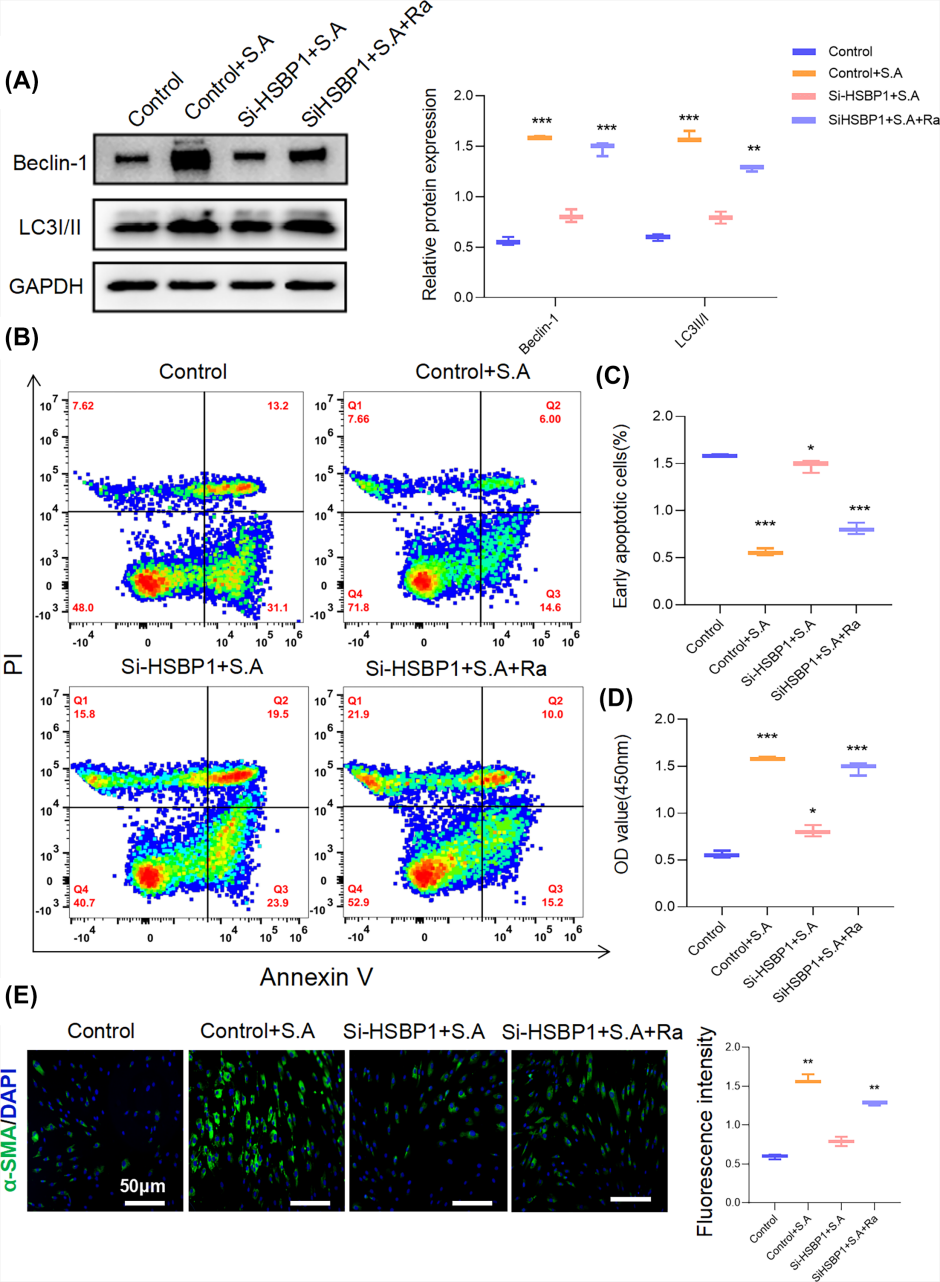
HSBP1‐mediated autophagy regulates *S. aureus*‐induced HFB fibrosis. (A) Representative western blot of Beclin1 and LC3 in fibroblasts pretreated with si‐HSBP1 with or without *S. aureus* (10^5^ CFU/mL) in the presence or absence of Rapmysin for 24 h. The protein levels were normalised to GAPDH levels. Graphs are presented as mean ± SD. Data were analysed using a two‐way ANOVA with Dunnett's multiple comparison test. **p < 0.01, ***p < 0.001, *n* = 3. (B‐C) The fibroblasts were treated with si‐HSBP1 with or without *S. aureus* (10^5^ CFU/mL) in the presence or absence of Rapmysin for 24 h, and the early apoptotic cells were detected by flow cytometry. Graphs were expressed as mean ± SD. *p < 0.05, ***p < 0.001, *n* = 3. (D) CCK8 was performed to detect the proliferation of normal fibroblasts pretreated with si‐HSBP1 with or without *S. aureus* (10^5^ CFU/mL) in the presence or absence of Rapmysin for 24 h. *p < 0.05, ***p < 0.001, *n* = 6. (E) Representative immunofluorescence microscopy demonstrating α‐SMA expression in fibroblasts pretreated with si‐HSBP1 with or without *S. aureus* (10^5^ CFU/mL) in the presence or absence of Rapmysin for 24 h. Scale bar:50 μm, Statistical analysis was performed using one‐way ANOVA with a Tukey's multiple comparison test, **p < 0.01, *n* = 6.

The levels of apoptosis and MFB transition in HFBs were measured in the absence and presence of rapamycin, a potent and specific mechanistic target of rapamycin (mTOR) inhibitor that also induces autophagy.^14^ The results indicated that the suppression of autophagy observed in *S. aureus*‐treated HFBs following the knockdown of HSBP1 via siHSBP1 was reversed upon the addition of an autophagy activator. These results suggest that HSBP1 plays a pivotal role in modulating autophagy in response to *S. aureus* infection and that exogenous activation of autophagy can overcome the inhibitory effects of HSBP1 knockdown on this process (Figure 6A). Consistently, the regulation of apoptosis as well as MFB transition by HSBP1 knockdown was rescued in the presence of an autophagy activator compared with that in control cells (Figure 6B–E).

## 4 DISCUSSION

HSs are pathological scars that occur after deep skin injury. After skin injury, fibroblasts are activated, leading to the initiation of abnormal proliferation, differentiation and ECM deposition.^15^ However, the mechanism of HS formation remains obscure. Our study found that the overloaded bacteria that colonise scars stimulate fibroblast proliferation and MFB transition.

In recent years, bacteria have received increasing attention in fibrosis‐related diseases. A study on intestinal bacteria revealed their driving role in liver fibrosis through the liver–gut axis.^16^ Another study by Muyiwa et al. reported that bacteria play an important role in the process of duct fibrosis in primary sclerosing cholangitis.^17^ In these studies, bacteria primarily activated fibrotic pathways by stimulating inflammatory responses.^18^, ^19^

In the current study, we utilised *S. aureus* as a representative of overloaded bacteria within scar tissue to stimulate fibroblasts. Our findings revealed that *S. aureus* directly promotes the proliferation and differentiation of these fibroblasts, ultimately inducing the increased secretion of collagen and VEGF. Unlike previous studies, our research reveals that the effect of bacteria on fibroblasts is mediated through HSBP1‐induced autophagy, rather than inflammatory responses, potentially opening a new direction in understanding the mechanism of scar formation.

Specifically, we observed an increase in HSBP1 levels in fibroblasts stimulated by *S. aureus*. Furthermore, inhibiting HSBP1 effectively prevented both the autophagy and fibrosis functions of these fibroblasts. Moreover, the preventive effect of fibrosis achieved by HSBP1 knockdown was reversed with the autophagy activator rapamycin.

HSBP1 is a member of the heat shock system, a conserved cellular mechanism that responds to extreme conditions, such as heat stress.^20^ Although bacterial stimulation also poses severe stress on cells, few studies have reported on the correlation between bacterial stimulation and the heat shock system in humans. One study in chickens showed elevated HSP70 expression in chicken embryos following infection with *S. aureus*, indicating that an increased level of HSP70 protein can be a useful indicator of infection caused by *S. aureus*.^21^ In our study, proteomics analysis revealed that HSBP1 expression increased significantly in *S. aureus*‐stimulated fibroblasts in vitro, which was associated with the fibrosis function of these fibroblasts. This finding might provide further evidence for the potential relationship between the heat shock system and bacterial stimulation.

Substantial evidence supports that the molecular function of HSBP1 is involved in cell growth and tumour differentiation. HSBP1 enhances the stem cell‐like characteristics in oral squamous carcinoma by boosting the presence of key stem cell markers, such as CD44, CD133, aldehyde dehydrogenase (ALDH) and SRY box‐containing gene 2 (SOX2). This process suggests that HSBP1 plays a significant role in transforming cancer cells to act more like stem cells^22^; Zhong et al. further reported that augmenting the expression of HSBP1 results in an upregulation of stem‐like characteristics in ovarian cancer cells.^10^ Interestingly, our research has uncovered a parallel function of HSBP1 in dermal fibroblasts. Specifically, when HSBP1 is overexpressed, it leads to a notable decrease in fibroblast apoptosis, accompanied by increases in both the proliferation and differentiation of these cells. These observations highlight the diverse roles that HSBP1 can play in regulating cellular behaviour across different tissue types. Indeed, previous investigations have largely overlooked the intricate mechanisms underlying HSBP1's ability to modulate cellular function. Our study sheds light on this gap by revealing that HSBP1 overexpression stimulates the expression of LC3 and Beclin‐1, two key markers of autophagy. This suggests that HSBP1 might be a key regulator of autophagy, an evolutionarily conserved process that plays a pivotal role in the maintenance of cellular homeostasis.^23^ Additionally, our results revealed that fibrosis regressed when autophagy was activated externally, even after reducing HSBP1 levels with gene knockdown. These results imply that HSBP1 enhances fibroblasts' performance by triggering autophagy. HSP70 empowers tumour cells to survive and thrive amidst persistent stressful conditions. A recent study revealed that blocking HSP70 activity leads to enhanced phosphorylation of Beclin‐1, a process mediated by the AMP‐activated protein kinase (AMPK).^24^ Given that HSBP1 functions as a negative regulator within the heat shock system, this observation hints at a close interplay between the autophagy and heat shock systems.

Indeed, the autophagy and heat shock systems are two crucial cellular defence mechanisms that safeguard cells against stress. Moreover, the coordinated and sequential activation and downregulation of the heat shock response and autophagy are paramount for maintaining optimal protein homeostasis in eukaryotic systems. This intricate interplay between these two systems is essential for cellular survival and function under stressful conditions.^25^ On a cellular level, autophagy contributes to reduced apoptosis and ensures a stable internal environment within the cell.^26^ Previous research has highlighted a notable connection between autophagy and scar formation. It is widely accepted that the hypoxic environment present in scars serves as a pivotal factor in triggering hypoxia‐inducible factor 1α (HIF‐1α) expression and subsequently activating autophagy.^27^ Elevated autophagy levels within scar tissue have been shown to enhance the expression of TGF‐β, a growth factor that can exacerbate scar progression. These findings suggest that autophagy plays a pivotal role in the development and severity of scarring.^28^ Our research revealed a notable elevation in autophagy levels upon bacterial stimulation, suggesting that bacteria, in addition to hypoxia, may serve as another important trigger for autophagy activation in scar tissue.

Autophagy facilitates the breakdown and disposal of intracellular macromolecules and organelles within lysosomes. HSP70 performs dual roles: it acts as a chaperone protein, assisting in the proper folding and assembly of other proteins, and it also functions as a lysosomal membrane stabiliser, safeguarding the integrity of lysosomes and their ability to degrade intracellular materials. Recent research has demonstrated that the cleavage of HSP70 can lead to lysosomal membrane permeabilisation and ultimately rupture,^29^ indicating that a decrease in HSP70 inhibits autophagy via the lysosomal pathway. In line with this, our research provides novel insights into the intricate interplay between autophagy and the heat shock system, potentially advancing our understanding of the functional changes that occur in fibroblasts within scar tissue. This knowledge may contribute to the development of more effective treatments for scarring.

A limitation of our study is that although we demonstrated that HSBP1‐regulated autophagy plays an important role in the activation of fibroblasts, an in‐depth investigation of how HSBP1 participates in the regulation of autophagy was not performed. A previous study revealed that HSBP1 serves as a novel interaction partner with FAK family kinase‐interacting protein of 200 kDa (FIP200) and autophagy‐related protein 13 (ATG13), two integral subunits of the unc‐51‐like autophagy‐activating kinase 1 (ULK) kinase complex, a pivotal regulatory element of the autophagy pathway. HSBP1 facilitates the assembly of these two components, thereby initiating the autophagic process.^30^ Given this evidence, it is reasonable to speculate that the same mechanism involving the interaction of HSBP1 with FIP200 and ATG13 may also underlie its role in modulating autophagy as observed in our study. In addition, the linear bacterial regulation of fibroblasts was disrupted when cells were treated with 106 CFU/mL *S. aureus*, suggesting that an increase in fibroblast apoptosis might not be the sole mechanism involved. Our future research should focus on identifying additional molecules that interact with HSBP1 and contribute to its autophagy‐related functions to provide a more comprehensive understanding of this complex regulatory network.

Currently, there are many modalities to treat HSs.^31^ Despite the availability of various treatment options, HSs remain a persistent clinical challenge. Our research has uncovered a novel mechanism, wherein an overabundance of bacteria in scar tissue directly promotes scar formation through HSBP1‐mediated autophagy. This discovery highlights HSBP1 as a promising new target for the management of scarring. However, these conclusions still need to be verified through in vivo experiments.

## AUTHOR CONTRIBUTIONS

Conceptualisation: XQW. Methodology: JRY, BY. Investigation: JRY, XQW, ZGM. Visualisation: BY, JRY. Funding acquisition: XQW. Project administration: XQW, JRY. Supervision: XQW. Writing—original draft: JRY, BY. Writing—review & editing: XQW.

## FUNDING INFORMATION

This work was supported by grants from the National Natural Science Foundation of China (Nos. 81671914 and 82472555).

## CONFLICT OF INTEREST STATEMENT

The authors declare that they have no conflicts of interest.

## Data Availability

Data are available in the main text or the supplementary materials.
